# Liquid biopsy and tumor heterogeneity in metastatic solid tumors: the potentiality of blood samples

**DOI:** 10.1186/s13046-020-01601-2

**Published:** 2020-05-27

**Authors:** Marco Russano, Andrea Napolitano, Giulia Ribelli, Michele Iuliani, Sonia Simonetti, Fabrizio Citarella, Francesco Pantano, Emanuela Dell’Aquila, Cecilia Anesi, Nicola Silvestris, Antonella Argentiero, Antonio Solimando, Bruno Vincenzi, Giuseppe Tonini, Daniele Santini

**Affiliations:** 1 Department of Medical Oncology, Campus Bio-Medico University of Rome, Álvaro del Portillo, 21, 00128 Rome, Italy; 2 Medical Oncology Unit, IRCCS-Istituto Tumori “Giovanni Paolo II” of Bari, 70124 Bari, Italy; 3 Department of Biomedical Sciences and Human Oncology, University of Bari ‘Aldo Moro’, 70124 Bari, Italy; 4 Department of Biomedical Sciences and Human Oncology, Section of Internal Medicine ‘G. Baccelli’, University of Bari Medical School, 70124 Bari, Italy

**Keywords:** Liquid biopsy, Tumor heterogeneity, Circulating tumor cells, Peripheral blood mononuclear cells, Circulating tumor DNA, microRNA

## Abstract

In a large number of cancer types, treatment selection depends on the presence of specific tumor biomarkers. Due to the dynamic nature of cancer, very often these predictive biomarkers are not uniformly present in all cancer cells. Tumor heterogeneity represents indeed one of the main causes of therapeutic failure, and its decoding remains a major ongoing challenge in the field.

Liquid biopsy is the sampling and analysis of non-solid biological tissue often through rapid and non-invasive methods, which allows the assessment in real-time of the evolving landscape of cancer. Samples can be obtained from blood and most other bodily fluids. A blood-based liquid biopsy can capture circulating tumor cells and leukocytes, as well as circulating tumor-derived nucleic acids.

In this review, we discuss the current and possibly future applications of blood-based liquid biopsy in oncology, its advantages and its limitations in clinical practice. We specifically focused on its role as a tool to capture tumor heterogeneity in metastatic cancer patients.

## Background

In the last decades, advances in precision medicine have radically changed the therapeutic scenario in medical oncology. The use and efficacy of such tailored therapies, including tyrosine kinase inhibitors (TKIs) and immune checkpoint inhibitors, often relies on the presence of specific tumor biomarkers, such as activating gene mutations or expression levels of specific proteins [1]. In many cases, however, these biomarkers are not uniformly present in all cancer cells, and such heterogeneity might hinder the therapeutic efficacy of tailored therapies [2].

Tumor heterogeneity refers to the coexistence of different biological, morphological, phenotypic and genotypic profiles, between tumors (inter-tumor heterogeneity) and within tumors (intra-tumor heterogeneity). It exists at multiple levels and may be present within different tumor regions or between primary cancer and metastases (spatial heterogeneity), or during the course of disease progression (temporal heterogeneity). The tumor microenvironment (TME), defined as the complex ecosystem in which cancer cells interact with non-cancerous cells, represents an additional source of intra-tumor heterogeneity. The TME includes proliferating tumor cells, the tumor stroma, surrounding blood vessels, and immune cells. In particular, the dynamic interplay between cancer and immune cells has become an issue of great interest. There is growing recognition that immunoediting, the process whereby the immune system can both counteract and promote tumor development, contributes to cancer heterogeneity and represents a potential source of biomarkers [3–6].

Tissue biopsy is the most widely used method for categorizing tumors and detecting biomarkers. However, it has a number of limitations: it is an invasive method; it is not always feasible or repeatable; it provides information limited to a single point in space and time, therefore failing to capture the complex tumor heterogeneity.

**Fig. 1:**
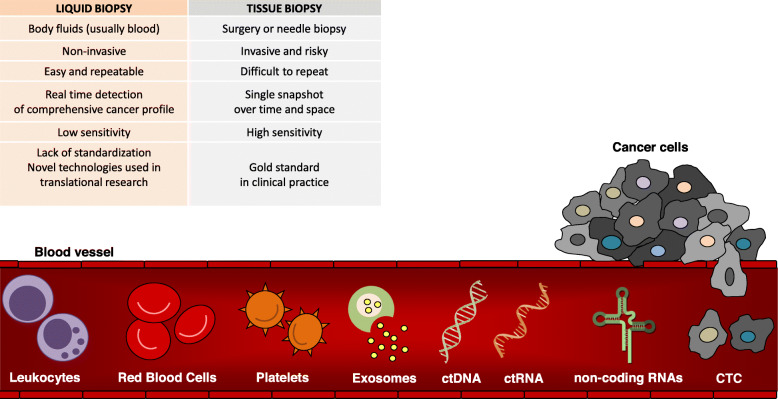
Liquid biopsy, non invasive and low-risk procedure, allows to monitor the changing and evolving landscape of cancer in real-time during the course of disease. In blood vessels, circulating tumor DNA (ctDNA), circulating tumor RNA (ctRNA), non-coding RNAs, circulating tumor cells (CTCs), and circulating leukocytes represent promising biomarkers to assess tumor heterogeneity and patients’ treatment response

To overcome these limitations, in recent years there has been an increasing development of liquid biopsy, defined as the sampling and analysis of non-solid biological tissue, such as blood and most other bodily fluids (e.g. urine, saliva, ascites, pleural effusion or cerebrospinal fluid). Liquid biopsy often represents a rapid and non-invasive alternative to tissue biopsy. Additionally, it allows for the longitudinal evaluation of cancer evolution. Blood-based liquid biopsy consists in the isolation and analysis of tumor-derived or tumor-associated components that circulate in the bloodstream (Fig. 1): circulating tumor cells (CTCs), circulating leukocytes, as well as tumor-derived circulating nucleic acids, such as cell-free circulating tumor DNA (ctDNA), microRNA (miRNA) and non-coding RNAs (ncRNAs) [7, 8].

In this review, we summarize the evidences on the role of blood-based liquid biopsy as a potential tool to capture tumor heterogeneity in metastatic cancer patients, and its current and future role in identifying biomarkers that might contribute to the therapeutic decision-making.

## Circulating tumor cells

The longitudinal, non-invasive, and comprehensive analysis of the genetically and phenotypically heterogeneous cancer cells is a major challenge for the modern oncologist. CTCs are defined as cancer cells found in the bloodstream. They are simultaneously shed both by the primary and metastatic sites, possibly providing a direct measure of spatial heterogeneity [9]. Given their rarity (in the range of 0.1–10 CTCs per mL of whole blood), a number of strategies have been developed to detect their presence in blood samples, based on either physical and morphological proprieties (e.g. nuclear irregularity, high nuclear/cytoplasmic ratio), or biological characteristics (e.g. the expression of the epithelial markers such as epithelial cell adhesion molecule, EpCAM, and cytokeratins) [10]. However, currently used CTC assays do not reach 100% of sensitivity and specificity for cancer detection. Indeed, patients with benign inflammatory colon diseases might harbor viable circulating epithelial cells that can be detected with current CTC assays [11]. Conversely, patients with epithelial cancers might present circulating cancer cells expressing mesenchymal rather than epithelial markers, because of epithelial-to-mesenchymal transition (EMT), a phenomenon associated to disease progression [12–14]. Moreover, it should be noted that CTC clusters are more difficult to detect with the aforementioned conventional methods, and yet they likely have higher metastatic potential compared to individual CTCs [15, 16]. Despite these limitations, CTCs have shown prognostic implications in a variety of cancer types, including breast cancer (BC), prostate cancer (PC), non-small cell lung cancer (NSCLC), colorectal cancer (CRC) and others [17]. Importantly, one of the main advantages of CTCs compared to other analytes of liquid biopsies is that CTCs can be cultured and expanded ex vivo to perform functional tests or subsequent single-cell sequencing analyses [18, 19].

### CTCs and spatial heterogeneity

A number of studies in different diseases have investigated CTCs as a way to gain insights in tumor spatial heterogeneity (Table 1).

**Table 1:** CTC and Spatial heterogeneity

Tumor type	Findings	References
Breast Cancer	Possible escape mechanism to endocrine therapy due to high percentage of ER negative CTC	[20]
	Discordance in HER2 status between primary and metastatic status influencing response to anticancer treatment	[21]
Prostate Cancer	AR signaling modification upon hormonal treatments influences outcomes	[22]
	Wnt activation leading to hormonal treatment failure	[23]
	CTC heterogeneity as indicator for first line treatment	[24]
	AR-V7 nuclear expression predicts better response to chemotherapy compared to AR signaling inhibitors	[25]
Colorectal Cancer	50% concordance in *KRAS* status between primary tumor and CTC as surrogate of spatial heterogeneity	[26]
	Differential phenotype of CTC from right and left side may explain different metastasization patterns	[27]
Hepatocellular Cancer	EMT of CTC relates with metastasization process	[28]

A different phenotype between primary tumor and CTCs can potentially predict poorer response to conventional anticancer therapy: for example, in metastatic BC (mBC), estrogen receptor (ER)-negative CTCs can be detected in patients diagnosed with ER-positive mBC [20] and HER2-positive CTC clones can be found in patients affected by HER2-negative mBC [21]. The presence of such CTC clones might anticipate failure to established therapies respectively targeting the estrogen axis and HER2.

Similarly, in metastatic castration-resistant PC (mCRPC) patients it is possible to identify CTCs with different levels of regulation of the androgen receptor (AR) signaling pathway [22]. RNA sequencing of individual CTCs showed heterogeneity in the expression of AR alterations, including splice variants, and in the expression of AR-independent pathways, such as glucocorticoid receptor and non-canonical Wnt signaling responsible for resistance to antiandrogen therapies [23].

Moreover, CTCs have shown a possible role as marker of spatial heterogeneity also in metastatic CRC (mCRC). The concordance of the *KRAS* status between primary tumors and CTCs varies between 50% [26] and 77% [24], supporting the existence of different clones within primary mCRC. Additionally, CTCs from left-side mCRC more frequently display a mesenchymal phenotype coherent with EMT, while CTCs from right-side mCRC show an apoptotic morphology [27].

EMT may be significantly involved in metastasization process of several cancer types; in fact, CTCs from patients affected by Hepatocellular Carcinoma (HCC) display epithelial phenotype at early stages, but they undergo mesenchymal transformation during spreading to metastatic sites [28].

Given the growing interest in the relationship between cancer and immune heterogeneity, immune checkpoint biomarkers have been analyzed on CTCs, in particular in metastatic NSCLC and mBC patients, who show high inter-individual heterogeneity of PD-L1 expression [29]. In particular, CTCs resulted to be more frequently PD-L1 positive in comparison with tissue samples in NSCLC, suggesting that CTCs may reflect spatial heterogeneity better than tissue biopsy [30] or alternatively that PD-L1 positive cells are more likely to acquire features consistent with CTCs.

Finally, a number of studies are exploring whether CTCs-derived information might guide treatment decisions. For example, quantification of the phenotypic heterogeneity of mCRPC CTCs [31] or their expression of nuclear AR splice variant 7 (AR-V7) [25] might help to guide the choice between AR signaling inhibitors and taxanes: lower CTC degree of heterogeneity is associated with better outcomes during AR Signaling Inhibitors (ARSI), while AR-V7 positive CTC predict with better outcomes during taxanes over ARSI.

### CTCs and temporal heterogeneity

Although not validated in clinical practice, both the CTCs count and their characterization are being explored as tools for monitoring the evolution of metastatic cancers as well as their sensitivity to anti-neoplastic drugs. For example, the reduction of the number of CTCs during treatment is associated with lower probability of disease progression, and longer progression-free and overall survivals in HER2-positive and HER2-negative mBC patients, but not in triple negative patients [32].

Several other examples of dynamic cellular changes that might be monitored over time with CTCs exists: for example, in mCRC patients it is possible to monitor the mutation status of *KRAS* in CTCs to anticipate changes in therapy [33]. Alternatively, next-generation sequencing (NGS) can be used in CTCs to assess multiple genes associated to resistance to therapies targeting the epidermal growth factor receptor (EGFR) [34]. Similarly, in EGFR-mutated NSCLC cancer progressing to anti-EGFR TKIs, a number of studies have evaluated the expression of resistance mutations and rearrangements [35], such the *EGFR* T790M secondary mutation [36] or MET amplification [37]. Finally, CTCs can be used to longitudinally assess the presence and intra-patient heterogeneity of *PIK3CA* mutations [38] which are associated to resistance to anti-HER2 therapies in mBC patients [39] (Table 2).

**Table 2:** CTC and Temporal heterogeneity

Tumor type	Findings	References
Colorectal Cancer	CTC *KRAS* status changes upon treatment and may potentially anticipate sensitivity to chemotherapy regimens	[33]
EGFR-mutated Non Small Cell Lung Cancer	Detection of acquired resistance mechanisms after first line EGFR-TKI treatment	[35, 36]
HER2-negative Breast Cancer	Assessment of PIK3CA during systemic treatment could inform about primary or acquired resistance	[38]

## Circulating leukocytes

The number, subsets, and molecular characteristics of leukocytes have been analyzed in cancer patients as prognostic and predictive biomarkers for several decades. Notoriously, the neutrophil-to-lymphocyte ratio has been proposed as an inflammatory biomarker elevated in patients with more advanced or aggressive diseases [40]. T cell receptor (TCR) profiling and surface immunoprofiling of circulating leukocytes are emerging powerful tools to detect the immunological cancer heterogeneity (Table 3).

**Table 3:** Tumor heterogeneity: potentiality of TCR profiling and circulating leukocytes

Tumor type	Findings	References
Pancreatic Ductal Adenocarcinoma	TCR features are correlated with survival in immunotherapy treated patients	[41]
Melanoma	TCR repertoire profiling is associated with immunotherapy response	[42–44]
	Baseline frequency of CD14 + CD16-HLA-DRhi monocytes, CD69 + MIP-1β + NK cells, and PD-1 + CD56+ T cells are potential predictors of clinical response in patients treated with immunotherapy	[45–47]
	The increase of central memory CD4+ T cells and the decrease of dysfunctional PD-1 + CD38hi CD8+ cells during immunotherapy are correlated with response.	[48, 49]
	Levels of circulating CD33 + CD11b + HLA-DR- myeloid derived suppressor and distinct CD4+ and CD8+ memory T cell subsets are correlated with survival of immunotherapy treated patients.	[46, 50]
Lung Cancer	TCR repertoires of PD-1+ CD-8+ lymphocytes are correlated with clinical outcomes of immunotherapy treated patients	[51, 52]
	Baseline percentage of HLA-DR monocytes and dendritic cells are correlated to immunotherapy response	[53]
Melanoma and Lung Cancer	Elevated frequencies of CD4 + Foxp3- T cells, at baseline and/or during immunotherapy, are associated with a higher risk of death	[54]

### TCR profiling of lymphocytes from cancer patients

The TCR is a polymorphic receptor dictating antigen specificity of the T-cell mediated immunity. TCR sequencing has been used as a tool to measure the heterogeneity of the T cells infiltrating tumor samples and of the immunogenic neoantigen burden [55–57]. In lung cancer patients, TCR spatial heterogeneity reflected the heterogeneity of the mutational landscape. In particular, the number of ubiquitous and regional TCRs correlated with the number of ubiquitous and regional non-synonymous mutations, respectively [58]. The role of TCR profiling in circulating lymphocytes is less clear. In fact, the TCR repertoires of tumor-infiltrating and matched peripheral lymphocytes only partially overlap [59, 60]. Nevertheless, tumor-specific T cells have been identified in the peripheral blood of cancer patients [61, 62]. Whether these repertoires might predict response to immunotherapy in metastatic cancer patients is still unclear. In melanoma patients, the peripheral TCR repertoire correlated with responses to checkpoint inhibitors [42–44], and similar results were reported for pancreatic ductal adenocarcinoma and other solid tumors [41]. Similarly, the evaluation of peripheral TCR repertoire of PD-1 + CD8+ lymphocytes also showed promising results as a non-invasive approach for selecting metastatic NSCLC patients who could benefit from immune checkpoint blockade [51, 52, 63].

### Immunoprofiling of peripheral leukocytes from cancer patients

With the technological advancements brought by flow cytometry first and mass cytometry after, our capability to identify rare subsets of circulating/ leukocytes has grown exponentially [64]. The predictive role of specific leukocyte subsets in patients undergoing anti-PD-1 and anti-CTLA4 immunotherapy has been investigated mostly in metastatic melanoma and lung cancer patients.

The baseline frequency of CD14 + CD16-HLA-DRhi monocytes [45], CD69 + MIP-1β + NK cells [46], and PD-1 + CD56+ T cells [47] were reported to be predictors of clinical response in metastatic melanoma patients treated with anti-PD-1 immunotherapy. In the same setting, the post-treatment increase of the specific subset of central memory CD4+ T cells, harboring the CD27 + FAS-CD45RA-CCR7+ phenotype, was associated to prolonged clinical responses [48]. Moreover, the decrease in the percentage of dysfunctional PD-1 + CD38hi CD8+ cells was also correlated with immunotherapy benefit [49]. In advanced lung cancer patients, baseline percentage of HLA-DR monocytes and dendritic cells also correlated to the response to PD-1 inhibitor therapy [53]. In both lung cancer and melanoma patients, elevated baseline frequencies of CD4 + Foxp3- T cells expressing PD-1 and/or lack of their significant down-modulation after PD-1 blockade resulted in a higher risk of death [54].

Finally, after treatment with anti-CTLA4 therapy, the levels of circulating CD33 + CD11b + HLA-DR- myeloid derived suppressor cells correlated with survival [50] in melanoma patients, as well as distinct CD4+ and CD8+ memory T cell subsets [46].

As more evidences accumulates, it emerges that cellular immunoprofiles and changes in TCR repertoires could predict responses to anti-PD1 and anti-CTLA4 therapies. It is yet unclear whether these biomarkers might represent alternative or complementary analyses in addition to more other markers for immunotherapy response (e.g. immunohistochemical analysis of PD-L1 or tumor mutation burden).

Although profiling of circulating leukocytes is a very promising tool, the absence of standardized protocols and the requirement of very sophisticated and expensive technologies to perform such analyses limit its use in the clinical setting.

## Circulating DNA

The discovery that blood-derived circulating-free DNA (cfDNA) contains tumor-specific genetic and epigenetic alterations has provided a solid ground to clinical usage of circulating tumor DNA (ctDNA) as a biomarker. Indeed, in the peripheral blood of cancer patients the amount of cfDNA is higher than in healthy subjects and it is partially composed by ctDNA directly released by tumor cells after apoptosis, necrosis or active secretion [65]. Due to the extremely low fraction of ctDNA, highly sensitive and advanced molecular detection technologies, as well as ctDNA-specific isolation methods are required.

The rapid development of new molecular technologies, like NGS and digital PCR (dPCR) has facilitated the clinical applications of ctDNA. In particular, dPCR is widely used for its low costs and high sensitivity that allows detecting mutations whose frequency is less than 0.1%. Nevertheless, PCR-based methods can only screen for known mutations, so their clinical applications are limited. At contrary, NGS platforms are less sensitive (detecting mutations with a frequency < 1%), but they are able to detect unknown genome-wide DNA mutations. Unfortunately, the routine use of NGS platforms in clinic is currently limited by their high costs. A frequently explored solution is the combination of the two techniques, NGS providing a first broad and exploratory view of mutation profile, with dPCR used to validate NGS results and to monitor the identified mutations over time, saving resources [66].

Overall, the quantitation and analysis of ctDNA can provide relevant clinical information about tumor burden, stage, vascularity, and therapy response (Table 4) [67, 68].

**Table 4:** Tumor Heterogeneity: significance of ctDNA

Tumor type	Findings	References
Breast Cancer	Identification of ER mutations in ctDNA not present in DNA from tumor biopsy	[69]
	ER mutations in ctDNA is associated with resistance to endocrine therapy	[70, 71]
	Identification of PIK3CA alterations in plasma-derived ctDNA	[72]
	PIK3CA ctDNA levels are associated with response to palbociclib and fulvestrant therapy	[73]
	HER2 mutation frequency predicts response to neratinib	[74]
	Association of ctDNA fraction and somatic copy number alterations with worse outcomes	[75, 76]
Non Small Cell Lung Cancer	Association of EGFR mutations with survival	[77, 79]
	Detection of EGFR mutations in ctDNA allows to identify patients eligible for anti-EGFR treatments (FDA-approved)	[78]
	Identification of EGFR mutations responsible of response to gefitinib	[79]
	Identification of EGFR mutation responsible of anti-EGFR therapy resistance (e.g. T790M)	[80]
	Longitudinal quantitative changes in ctDNA correlate with therapeutic response	[82]
Colorectal Cancer	ctDNA analysis allows to identify KRAS, BRAF, APC, PIK3CA, EGFR and NRAS mutations helping clinicians’ treatment strategy choice	[83, 88]
	Detection of EGFR and APC mutations in ctDNA to track clonal evolution and therapy response	[84, 85]
	KRAS mutations in ctDNA can be detected before radiological relapse	[87]
Castration Sensitive Prostate Cancer	ctDNA provides complementary information to a prostate needle biopsy and could be used to guide management strategies	[89]
	Detection of AR gene alteration to monitor treatment response or resistance	[90, 91]

### The potential role of ctDNA in metastatic disease

Several evidences reported that somatic alterations in commonly mutated genes can be identified in ctDNA and, in some cases, ctDNA allows to detect additional mutations not found by sequencing of a single metastatic lesion. In this regard, Chu et al. showed that the sequencing of ctDNA through NGS systems identifies mutations in *ESR1*, the gene encoding the ER, not detected in the corresponding biopsy of a metastatic lesion in mBC patients [69]. These results support the increasingly evidences of heterogeneity between different metastatic sites within the same patient. In addition, ESR1 mutations found by dPCR in ctDNA of mBC patients were associated with resistance to endocrine therapy suggesting that ctDNA represents a more sensitive strategy to monitor treatment efficacy [70]. dPCR sequencing of baseline plasma DNA from SoFEA and PALOMA-3 trials identified ESR1 mutations that predicted resistance to exemestane and sensitivity to fulvestrant, helping clinicians to choose the best treatment strategy for patients [71]. Similarly, alterations in phosphatidylinositol 3-kinase (PI3K) *PIK3CA* gene, the most commonly mutated oncogene in BC, have been identified in ctDNA [72]. Since *PIK3CA* mutational status can change upon disease recurrence, ctDNA analysis might provide an excellent tool to monitor sub-clonal changes in real-time. Indeed, in the PALOMA-3 study a drop in *PIK3CA* ctDNA levels after 15 days of therapy with fulvestrant and palbociclib, strongly predicted PFS [73]. Notably, ctDNA is currently gaining momentum not only in the endocrine therapy setting, but also in detecting HER2 mutations. In particular, ctDNA analysis of HER2^mut^ variant allele frequency demonstrated to be predictive of response to neratinib with sensitivity of 79% and a specificity of 100%, when compared to tumor tissue analysis [74]. Finally, in triple negative mBC patients, a ≥ 10% ctDNA fraction and the presence of copy number gain or amplification at specific loci was associated with significantly worse outcomes [75, 76].

Several studies reported the detection of EGFR activating mutations in ctDNA of patients with advanced NSCLC [77, 78]. The multicenter ASSESS study demonstrated that ctDNA is a feasible sample type for real-world EGFR mutation testing in metastatic NSCLC patients [79]. In 2015, EMA approved the use of ctDNA for EGFR mutation assessment. A year later, FDA approved COBAS EGFR Mutation Test v2 (Roche Molecular Systems, Inc.) for the detection of EGFR mutations in liquid biopsy, when tissue biopsy is not available, to identify metastatic NSCLC patients eligible for anti-EGFR treatment. Importantly, EGFR-T790M mutation, the most frequent alteration associated with TKI resistance, can be detected in ctDNA as an alternative to tumor DNA derived from a tissue sample [80]. Although the concordance is not full, ctDNA can replace the solid biopsy when the last is not feasible, it can be repeated several times and represents an excellent way for monitoring the treatment with anti-EGFR targeted therapies and for identifying novel resistance mutations [81]. Moreover, in metastatic EGFR mutated NSCLC patients, therapeutic response significantly correlate to the longitudinal quantitative changes in plasma ctDNA [82].

ctDNA has demonstrated a particular utility in monitoring treatment response and identifying mechanisms of resistance in metastatic CRC (mCRC). In particular, a high concordance between the mutational status of *KRAS*, *NRAS* and *BRAF* in the tissue and in ctDNA of CRC patients was found [83]. Interestingly, ctDNA analyses often detected *KRAS* mutations not detected in the surgical specimen [84]. There are evidences that ctDNA analysis could inform about clonal heterogeneity and subclonal changes in real time [85, 86]. In this regard, Siravegna et al. demonstrated that ctDNA detection allowed to track clonal evolution during therapies with anti-EGFR antibodies. In particular, time-course profiles of ctDNA of patients treated with cetuximab and panitumumab revealed that mutant RAS clones, which rise in blood during EGFR blockade, decline upon withdrawal of EGFR-specific antibodies. In this way, EGFR inhibitor can be rechallenged leading again to patients’ response [84]. In addition, longitudinal analysis of ctDNA showed that several mutations rapidly emerge during EGFR blockades, often before radiological relapse [87]. Recently, Kato S et al., reported that 79% of analyzed advanced CRC patients presented ctDNA genomic alterations in *TP53* (51% of patients), *KRAS* (34%), *APC* (27%), *BRAF* (16%), *PIK3CA* (16%), and *EGFR* (15%) genes. The authors showed that ctDNA could be helpful for clinicians in the definition of the most appropriate treatment for patients; indeed patients who received the matched targeted-therapy showed better responses compared with patients who received unmatched therapies [88].

ctDNA has demonstrated to be a promising source of clinically relevant information also in PC. A recent study demonstrated that primary tissue and ctDNA share relevant somatic alterations, suggesting that either is suitable for molecular subtyping in de novo metastatic castration-sensitive PC (mCSPC) [89]. Other evidences demonstrated that mutations in the AR gene can be effectively detected in ctDNA of mCRPC patients and provide insights into treatment response and resistance [90, 91].

In conclusion, the determination of EGFR mutations in ctDNA to guide anti-EGFR treatment in NSCLC patients is the first and so far only approved ctDNA assay. The main limitation in this field is related to the low abundance of ctDNA, whose detection requires advanced molecular platforms (NGS or dPCR) still too expensive to be routinely used in the clinics. Nevertheless, the continuous development of cheaper high-throughput sequencing techniques will most likely consolidate the evaluation of ctDNA in liquid biopsies as a promising tool in the next future [66].

## Circulating RNA

The development of RNA-based biomarkers is seemingly less successful compared to the other analytes of blood-based liquid biopsies, such as CTCs and ctDNA. Their use is in fact limited by the risk of contamination during sample isolation extraction, which is also present in the case of ctDNA, but also by their higher instability and lower abundance. However, recent studies have shown a broad potential that, if validated, could have important implications in future clinical practice [92–95]. Circulating RNA, for instance, may help clinicians to track therapy response or resistance that could reflect tumor heterogeneity.

Cancer cells can release RNA into the bloodstream through different mechanisms, some of which are mediated by microvesicles, as exosomes. Exosomes are cell-derived extracellular vesicles released by different cell types, including immune cells and cancer cells. They have a key role in cell-to-cell communication and, more recently, are coming to light as players of tumor-specific process such as proliferation and progression [96–98].

Circulating RNAs might provide real-time information on cancer-related events and could have a prognostic role. Among these, circulating mRNAs have been demonstrated to be a useful biomarker in monitoring tumor progression, especially in PC. In particular, the presence of AR splice variant 7 (AR-V7) transcripts in mCRPC patients has been correlated with shorter time to treatment failure. Moreover, AR-V7 RNA showed prognostic value in estimating the OS of these patients [99].

However, due to their higher stability, non-coding RNAs are significantly more abundant than mRNAs in the bloodstream. Therefore, in the last years the research has focused on non-coding RNAs and their role as biomarkers in metastatic cancer diseases.

### microRNA

Among non-coding RNAs, miRNAs are the best promising non-invasive biomarker due to the development of NGS technologies that have enabled the sequencing of the complete miRNA profile. Indeed, several studies identified miRNA as potential biomarkers, useful to monitor cancer progression, patients’ outcomes and the development of chemo-resistance [100] (Table 5). However, although miRNAs have been regarded as promising biomarkers for many years, they have never entered the clinics.

**Table 5:** Tumor Heterogeneity: significance of miRNAs

Tumor type	Findings	References
Breast Cancer	Upregulation of miR-21, miR-23b, miR-200b, miR-200c levels; miR-23b and miR-190 correlated with low PFS in de novo metastatic patients; high levels of miR-200b predicted decreased OS in the HER2-negative subgroup	[101]
Colorectal Cancer	Upregualtion of miR-103 levels were associated with lymph nodes metastases and advanced disease	[102]
	Upregulation of miR-29a	[103]
	miR-203 and miR-141 expression discriminated metastatic from early stage patients	[104]
	miR-21 correlated with liver metastases and TNM stage and was associated with worse OS and disease free survival	[105]
	Decreased levels of miR-1914-3p and miR-1915-3p were found in chemoresistant patients	[106]
Non Small Cell Lung Cancer	High expression level of exosomal miR-222-3p, miR-23b-3p, miR-10b-3p and miR-21-5p were associated with poor OS; miR-21-5p correlated with liver metastases and TNM stage.	[107, 108]
	Lower expression of exosomal miR-146-5p was found in cisplatin resistant patients and was associated to short PFS	[109]
Pancreatic ductal adenocarcinoma	High expression of miR-155-5p was correlated with chemoresistance and poor prognosis in patients receiving gemcitabine treatment	[110]

MiRNAs are endogenous, single-stranded, non-coding small RNAs with length of about 22 nucleotide that exert several cellular biological functions inhibiting their target genes [111]. Several overexpressed miRNAs, called oncomiRs, are involved in tumor onset and metastasis, instead those that decreased in cancer patients were considered tumor suppressors [112].

A recent study reported that the expression levels of different specific miRNAs (miR-21, miR-23b, miR-200b, miR-200c) were found higher in metastatic compared to early BC patients. In addition, miR-23b and miR-190 were correlated with low PFS in de novo metastatic BC patients, whereas high levels of miR-200b were associated to decreased OS in the HER2-negative subgroup [101].

The ability of circulating miRNAs to discriminate patients with lymph nodes and/or distant metastasis has been also examined in advanced stages of CRC. In particular, upregulated serum levels of miR-103 were associated with lymph node metastasis and advanced stage of CRC [102]. Similarly, higher serum levels of miR-29a were found in metastatic CRC patients compared to non-metastatic patients. However, this miRNA did not demonstrate a sufficient accuracy in discriminating these groups of patients, showing a sensitivity of 75% and a specificity of 75% [103].. Another study showed instead that plasma expression of miR-203 and miR-141 are able to discriminate between advanced from early stage CRC patients with a good performance [104]. Finally, Tsukamoto et al. have recently reported the levels of miR-21 are elevated in serum exosomes, primary tumor tissues, and liver metastasis tissues from CRC patients. In particular, exosomal miR-21 showed a correlation with liver metastases and TNM stage and was associated with worse OS and disease free survival [105].

Several evidences showed a potential role of circulating miRNA in identifying NSCLC patients with aggressive advanced disease. Indeed, elevated expression levels of miR-222-3p, miR-23b-3p, miR-10b-3p, and miR-21-5p in serum exosomes were associated with poor OS in NSCLC patients. Among these miRNAs, exosomal miR-21-5p overexpression was also associated with presence of liver metastasis and TNM stage [107, 108].

Since cancer heterogeneity is ultimately associated to drug resistance following prolonged treatments, circulating miRNAs could represent potential biomarkers useful to predict drug resistance and select effective treatment strategies. One example is miR-155-5p, whose expression is directly correlated with chemo-resistance and poor prognosis in pancreatic ductal adenocarcinoma patients receiving gemcitabine [110]. Another example is represented by the evidence of lower levels of miR-1914-3p and miR-1915-3p in the plasma of chemo-resistant CRC patients compared to responders [106]. Finally, lower levels of miR-146-5p are found in serum exosomes of cisplatin-resistant NSCLC patients and they are associated to shorter PFS [109].

Circulating miRNAs could represent non-invasive biomarkers, useful not only for large screening programs for tumors with higher sensitivity, but also to dynamically monitor tumor progression and treatment response [100]. Unfortunately, the complexity of RNA biomarker assays and data interpretation contribute to their low success rate and slow down their use into the clinical practice.

### The panorama of other non-coding RNAs

Besides the well-known miRNAs, there are other species of non-coding RNAs that are currently emerging as candidate biomarkers assessable in blood-based liquid biopsies, including long non-coding RNAs (lncRNAs), circular RNAs (circRNAs) and PIWI-interacting RNAs (pi-RNAs).

LncRNAs activate or silence the expression of genes regulating chromatin state, mRNA splicing, transport and translation of RNAs, act as competing endogenous RNAs and influence protein modification [113–115]. Intriguingly, among the target loci of lncRNA there are well-known genomic regions with oncogenic or tumor-suppressive functions, suggesting a key role of these RNAs in the control of cancer progression [116].

For example, the aberrant expression of the lncRNA HOTAIR (HOX antisense intergenic RNA) is associated to tumor proliferation, angiogenesis, progression, drug resistance and worse prognosis [117]. Numerous evidences have focused the attention on the potential role of HOTAIR as a circulating marker and therapeutic target is solid tumors [118]. In this regard, Li et al. demonstrated that high levels of HOTAIR were associated to tumor recurrence, radio-resistance and shorter OS in cervical cancer [119].

Similarly, in metastatic HCC patients, plasma levels of lncRNAs XLOC_014172 and LOC149086, allowed to discriminate, with high performance patients with metastatic disease from patients without secondary lesions [120]. Similarly, Wang et al. showed that serum lncRNA-p21 decreased in liver metastatic cancer patients, respect to non-metastatic HCC patients [121].

Several evidences showed the potential prognostic value of some lncRNAs also in CRC. Among these, higher exosomal levels of the oncogenic 91H were found in CRC patients’ serum and these levels were associated with metastatic disease and tumor recurrence [122]. Similarly, higher serum levels of exosomal CRNDE-h were observed in metastatic CRC patients compared to non-metastatic patients and were positively associated with poor OS [123]. Another lncRNA correlated with outcome in metastatic CRC is GNAT1–1, whose serum levels were decreased in patients with worst prognosis [124].

LncRNAs could represent important biomarkers also to monitor drug resistance that could occur in distinct tumor clones, helping clinicians in choosing patients’ best treatment. Increased lncRNA XIST levels, for instance, were found in serum of CRC patients nonresponding to 5-FU [125]. Similarly, reduced serum lncRNA MEG3 expression identified CRC patients resistant to oxaliplatin-based chemotherapy [126].

Circular RNAs (circRNA) are another class of non-coding RNAs that could be implicated with several human diseases, including cancer [127]. CircRNAs are closed circular one-stranded RNAs that act as competing endogenous RNA sponges modulating the activity of their targets (miRNAs, proteins and RNAs) [128]. Specific circRNAs have been detected in high quantities in blood of patients with different advanced solid tumors representing potential biomarkers useful to monitor tumor progression and response to treatments in metastatic stage [129]. In HCC patients, for instance, high serum expression of circ-ZEB1.33 was associated to tumor progression and was also related to patients’ OS [130]. Other circRNAs were associated to tumor stage and presence of metastases in gastric cancer (GC) patients. In particular, Hsa_circ_0000190 was found downregulated in GC patients’ plasma and was correlated to the presence of metastasis, tumor stage and CA19.9 levels [131]. Also in GC patients, decreased expression of plasma hsa_circ_0000745 was associated to TNM stage [132]. Recently, a key role of circRNA was found also in metastatic urothelial cancer patients, where high levels of circPRMT5 in serum exosomes correlated with the presence of metastasis and tumor stage [133].

PiRNAs regulate gene expression, ensure genome integrity of germline cells and control developmental timing. Aberrant piRNA expression was found in blood and is currently being associated to cancer progression and metastases [134]. Recently, a study demonstrated the positive correlation between piR-54,265 levels and tumor stage in CRC patients, with highest levels in the metastatic setting. Moreover, piR-54,265 lower serum levels were also predictive of chemotherapy response and potentially useful to select CRC patients who benefit from chemotherapy [135].

## Conclusions

Although blood-based liquid biopsy has shown a huge potential for different purposes in several tumor types, it is validated in clinical practice only for few selected uses. After its approval for the detection of EGFR mutations in NSCLC, it was extensively used to determine the T790M mutation, the main mechanism of resistance to first and second generation TKIs. Thus, many patients avoided additional tissue biopsy and a fraction of them was able to receive an otherwise inaccessible treatment. Its use declined when osimertinib, a third generation TKI, became the standard of care as frontline treatment in EGFR-mutant NSCLC. Nevertheless, this experience represents the proof-of-concept of the potentiality of liquid biopsy to change and improve clinical practice.

To date, liquid biopsy cannot be considered a replacement for tissue biopsy, which remains the standard and undisputed method for the diagnosis and biomarkers detection of all solid tumors, but it has undoubted advantages: it is a non-invasive, rapid, easy, repeatable and real-time test. It can provide a great amount of information, and it is potentially superior to tissue biopsy in its capability to sample tumor heterogeneity especially in the metastatic setting.

Liquid biopsy is usually less expensive than tissue biopsy, but in the case of repeated tests, the cumulative costs can be the same or higher. In addition, some of the technologies and molecular protocols used to detect analytes are very sophisticated and expensive; many of them need to be standardized and require further studies for clinical validation. The other most important barrier preventing the implementation of liquid biopsy in clinical practice is represented by its relatively low accuracy rate [136]. The management of small amounts and easily degradable materials requires extremely sensitive and specific methods. Both circulating tumor cells and DNA are relatively rare compared to the number of other molecules found in a blood sample. Accurate tumor information can therefore be obtained only when the abundance of CTCs or cfDNA is greater than specific thresholds, and a significant number of cancer patients do not meet this criterion [67, 137]. A committee of experts conducted a review of the published clinical ctDNA tests and concluded that the current clinical efficacy of liquid biopsy techniques is very limited [138].

To determine its effectiveness and clinical utility, liquid biopsy requires further and large-scale prospective studies. However, based on available evidence, it appears to have several potential applications: cancer screening and early diagnosis; estimation of the risk for metastatic relapse or metastatic progression; prediction of prognosis; longitudinal monitoring of disease progression and response to treatment; identification of therapeutic targets and resistance mechanisms.

In particular, thanks to the multiplicity of analytes that can be identified and the repeatability of the test, liquid biopsy represents an accessible tool to the decode both the spatial and temporal tumor heterogeneity. In the next future, it may significantly and non-invasively contribute to the clinical management and therapeutic decisions for cancer patients in the era of precision medicine.

## Data Availability

Not Applicable.

## Abbreviations

CTCs: Circulating Tumor Cells
ctDNA: circulating tumor DNA
ctRNA: circulating tumor RNA
cfDNA: *c*irculating free DNA
miRNA: microRNA
non-coding RNA: ncRNAs
EMT: *Epithelial*-to-mesenchymal transition
BC: Breast Cancer
PC: Prostate Cancer
CSPC: Castration Sensitive Prostate Cancer
CRPC: Castration Resistant Prostate Cancer
AR: Androgen Receptor
ARSI: AR Signaling Inhibitors
EGFR: Epidermal Growth Factor Receptor
TCR: T Cell Receptor
HLA: Human Leukocyte Antigen
ER: Estrogen Receptor
NSCLC: Non Small Cell Lung Cancer
CRC: Colorectal Cancer
GC: Gastric Cancer
PFS: Progression Free Survival
OS: Overall Survival
NGS: Next-generation sequencing
lnRNAs or lincRNAs: long non-coding RNAs
circRNAs: circular RNAs
pi-RNAs: PIWI-interacting RNAs
