# Correction to: Liquid biopsy and tumor heterogeneity in metastatic solid tumors: the potentiality of blood samples

**DOI:** 10.1186/s13046-020-01615-w

**Published:** 2020-06-24

**Authors:** Marco Russano, Andrea Napolitano, Giulia Ribelli, Michele Iuliani, Sonia Simonetti, Fabrizio Citarella, Francesco Pantano, Emanuela Dell’Aquila, Cecilia Anesi, Nicola Silvestris, Antonella Argentiero, Antonio Giovanni Solimando, Bruno Vincenzi, Giuseppe Tonini, Daniele Santini

**Affiliations:** 1 Department of Medical Oncology, Campus Bio-Medico University of Rome, Álvaro del Portillo, 21, 00128 Rome, Italy; 2 Medical Oncology Unit, IRCCS-Istituto Tumori “Giovanni Paolo II” of Bari, 70124 Bari, Italy; 3 Department of Biomedical Sciences and Human Oncology, University of Bari ‘Aldo Moro’, 70124 Bari, Italy; 4 Department of Biomedical Sciences and Human Oncology, Section of Internal Medicine ‘G. Baccelli’, University of Bari Medical School, 70124 Bari, Italy

**Correction to: J Exp Clin Cancer Res 39, 95 (2020)**

**https://doi.org/10.1186/s13046-020-01601-2**

Following publication of the original article [1], the authors identified an error in the author name of Antonio Giovanni Solimando.

The incomplete author name is: Antonio Solimando

The complete author name is: Antonio Giovanni Solimando

The author group has been updated above and the original article [1] has been corrected.
